# Mobile genetic elements and genome evolution 2014

**DOI:** 10.1186/1759-8753-5-26

**Published:** 2014-11-18

**Authors:** Parmit Kumar Singh, Guillaume Bourque, Nancy L Craig, Josh T Dubnau, Cédric Feschotte, Diane A Flasch, Kevin L Gunderson, Harmit Singh Malik, John V Moran, Joseph E Peters, R Keith Slotkin, Henry L Levin

**Affiliations:** Eunice Kennedy Shriver National Institute of Child Health and Human Development, National Institutes of Health, Bethesda, MD USA; McGill University and Genome Quebec Innovation Center, Montreal, Canada; School of Medicine, The Johns Hopkins University, Baltimore, MD USA; Howard Hughes Medical Institute, Chevy Chase, MD USA; Cold Spring Harbor Laboratory, Cold Spring Harbor, Cold Spring, NY USA; Department of Human Genetics, University of Utah School of Medicine, Salt Lake City, UT USA; Department of Human Genetics, University of Michigan, Ann Arbor, MI USA; Illumina Inc, San Diego, CA USA; Division of Basic Science, Fred Hutchinson Cancer Research Center, Seattle, WA USA; Department of Microbiology, Cornell University, Ithaca, NY USA; Department of Molecular Genetics, The Ohio State University, Columbus, OH USA

## Abstract

The Mobile Genetic Elements and Genome Evolution conference was hosted by Keystone Symposia in Santa Fe, NM USA, 9 March through 14 March 2014. The goal of this conference was to bring together scientists from around the world who study transposable elements in diverse organisms and researchers who study the impact these elements have on genome evolution. The meeting included over 200 scientists who participated through poster presentations, short talks selected from abstracts, and invited speakers. The talks were organized into eight sessions and two workshops. The topics varied from diverse mechanisms of mobilization to the evolution of genomes and their defense strategies against transposable elements.

## Introduction

Transposable elements (TEs) are powerful drivers of evolution. TEs constitute the majority of genomic DNA in many eukaryotes, and they dramatically shape genetic content by causing mutations, rearrangements, and sequence duplications. Of increasing significance is the link between these transposon-mediated mutations and disease. Recent advances in the study of TEs motivated Keystone Symposia to host the conference on Mobile DNA and Genome Evolution in Santa Fe, NM, USA, 9 March through 14 March 2014. The topics discussed at the conference often relied on recent innovations in high throughput sequencing and genome analysis. Results presented include discoveries of cellular systems that inhibit transposon activity, mechanisms of TE activity, evolutionary impact of TEs, and connections between TE activity and disease. Also included were intriguing results that documented TE activity during neurogenesis and aging. The symposium fostered ties between scientists in the field of transposon biology with the experts of genome evolution and analysis. The TEs and hosts represented in this meeting included a variety of examples from eubacteria, archaea, protists, plants, fungi, and animals. The meeting featured two keynote addresses by scientists who have made particularly important discoveries in the field of transposable elements.

### Keynote addresses

Allan Spradling (HHMI/Carnegie Institute, USA) opened the meeting with a keynote address entitled *Transposon Regulation and Genome Evolution.* He spoke on the role of TEs in regulation and genome evolution. He emphasized that the different insertion patterns of TEs reflect different transposition mechanisms and evolutionary strategies. In his role as director of the *Drosophila* Gene Disruption Project, Dr. Spradling examined the integration patterns of the TEs used in the project: *Minos*, *piggyBac*, and P elements. By dividing the genome into large numbers of equally sized segments, the integration of *Minos*, a Tc1/*mariner*-related element from *Drosophila hydei*, is found to be largely random, while *piggyBac*, from the cabbage looper moth, has a broad integration profile in *Drosophila melanogaster* that features integration in the promoter regions of genes. The focus of the talk then shifted to the P element, which displays a highly biased integration pattern that favors promoter sequences. Inserts into just 100 genes account for 40% of all integration events. In searching for a mechanism responsible for P element insertion in specific genes, Dr. Spradling found that in cultured cells the promoters with the highest level of integration events contained binding sites for the origin recognition complex. These data argue that P elements integrate at origins of replication. Dr. Spradling proposed the appealing model that coordination of transposition with DNA replication could increase P element copy number. Excision from recently duplicated DNA coupled with integration into unreplicated DNA would cause an overall increase in copy number because the excised copy could be repaired by homologous recombination using the replicated sequence as template. An additional feature of the model is transposition across replication forks would result in local transposition, a common feature of many TEs that remains unexplained. Regardless of whether integration is local, transposition from replicated into unreplicated DNA would result over time in the accumulation of P elements in late replicating DNA, much of which is heterochromatinized. This trend has been observed for P elements.

The DNA of *Drosophila* larval salivary glands, like many other types of polyploid cell DNA, is highly replicated through a process known as endoreduplication. However, a longstanding mystery is the nature of genome regions that are not fully replicated in polytene cells because they are lower in copy number than flanking sequences. Dr. Spradling examined the replication status of DNA isolated from salivary glands. By applying methods of high throughput sequencing he identified more than 100 regions of DNA from salivary glands that appeared to be under-replicated, compared to DNA from diploid cells. This was many more sites of under-replication than was previously known. Analysis of the sequence reads for deletions revealed a surprising correlation with the DNA found to have low copy number. Dr. Spradling made the provocative proposal that replication forks that stall or fail to complete replication, break and are repaired to generate deletions with diverse breakpoints that are responsible for the copy number changes. He suggested that this process generates genetic diversity in these regions in polytene cells that could be advantageous and that the generation of somatic alterations by incomplete replication might be widespread among polyploid cells in diverse organisms.

Haig Kazazian (Johns Hopkins University School of Medicine, USA) followed with a keynote presentation entitled *Biology of Human Retrotransposons: A Potential Role for Retrotransposition in Tumorigenesis?* Dr. Kazazian highlighted the role of retrotransposition in human disease. He described how in 1985 his studies of hemophilia led to the discovery of two spontaneous insertions of a LINE-1 (L1) element in the *Factor VIII* gene that resulted in disease. A few years afterwards Dr. Kazazian identified another disease-causing insertion of L1 that in this case was in the dystrophin gene. This insertion was unusual in that it carried non-L1 sequence 3′ to the L1, a so-called 3′ transduction event. These findings represented the first cases of disease that could be directly associated with TE integration. Subsequent studies have identified 101 cases of disease resulting from *de novo* retrotransposition events: 25 caused by L1 insertion, 61 due to Alu, 10 resulted from SVAs, four due to L1 poly (A) sequence transduction, and 1 processed pseudogene.

The sequences of the disease-causing L1s ultimately enabled Dr. Kazazian to isolate the source copies of L1. His development of genetic assays for transposition in cultured cells demonstrated that the source elements were highly mobile copies of L1. The transposition assay was used to test the activity levels of many L1 elements and these results demonstrated there is just one family of L1s that retains activity. Dr. Kazazian coupled this information with the reference genome sequence in estimating the average human genome has 80 to 100 active copies of L1. While this estimate was based on the reference genome Dr. Kazazian surmised that the sequence and position of active L1s could vary greatly between individuals. This assumption was borne out using PCR methods that specifically detect human families of L1. Dr. Kazazian assayed 16 individuals with this new PCR method and discovered 367 non-reference insertions of L1. In addition, his analysis of sequence from the 1,000 Genomes Project identified another 1,016 L1 insertions not found in the reference genome. These data together with results from other labs clearly indicate L1 elements are highly polymorphic in the human population.

Dr. Kazazian has now turned his attention to L1 transposition in somatic cells. With L1-targeted resequencing of colorectal tumor DNAs and matched normal DNAs, he found that certain cancers had large numbers of L1 insertions. Numerous genes associated with tumorigenesis had insertions suggesting somatic retrotransposition may play an active role in colorectal cancer. To test this possibility, Dr. Kazazian is currently analyzing precancerous tissues, including colorectal polyps, and comparing their L1 content to tumors and normal tissue from the same individuals. Many somatic insertions of L1s in colorectal tumors can be identified in the precancerous polyps, indicating the insertions occurred early in the development of the tumors. Dr. Kazazian is also studying precancerous lesions in esophagus resulting from acid reflux. While this study is ongoing, Dr. Kazazian reported that L1 insertion numbers were higher in the precancerous tissue than in the normal samples. Importantly, the esophageal cancers had greater numbers of L1 insertions than the precancerous lesions. These data argue L1 is active in somatic tissue and that the numbers of L1 insertions parallel the conversion of normal tissues into precancerous cells and finally into cancerous tumors. Dr. Kazazian is currently examining the positions of these insertions to assess whether L1 may contribute to the progression of cancer. In other studies Dr. Kazazian is studying the L1 insertions in metastases. Here, he reports that specific insertions in source tumors can be identified in metastases. These data suggest that L1 insertion sites could be used as biomarkers that could allow clinicians to determine the progression of primary tumors and metastases.

### Session 1: transposon applications and human health

Gerald Schumann (Paul Ehrlich Institute, Germany), explored how reprogramming to create human induced pluripotent stem cells (hiPSCs) and their subsequent cell culture induced genomic changes mediated by the mobilization of endogenous TEs. In particular, his lab analyzed the genomes of eight different hiPSC lines generated by Sleeping Beauty or lentiviral-based introduction of reprogramming genes, for the presence of endogenous *de novo* retrotransposition events that were absent from the respective parental cells. Both up-regulation of TE activity and concomitant demethylation of endogenous L1 elements were observed upon induction. Retrotransposon capture-sequencing (RC-Seq) was used to discover insertion sites within the host genome. Their experiments showed that endogenous L1-mediated retrotransposition is highly dynamic during reprogramming and hiPSC cultivation. Future in-depth experiments are required to assess whether this phenomenon alters the phenotype of hiPSC derivatives sufficiently to impact their use in medical or research applications.

Frederic Bushman (University of Pennsylvania, USA) explored the role of the human virome in human health. His lab explored the diversity of the virome within the gut across a dozen different individuals. They assessed longitudinal evolution of the gut virome by collecting a series of samples over 2.5 years from the same individuals. After sequencing and assembling more than 56 billion bases from the study, they identified 478 viral contigs of mostly bacteriophage origin. About 80% of the contigs were present throughout the longitudinal study, but a subset of contigs, as exemplified by elements of the circular Microviridae genome, showed rapid evolution with a near 4% speciation divergence over the 2.5-year study. This rapid divergence occurred through a number of different mechanisms including steady substitution, use of Diversity Generating Retroelements (DGRs, Jeffrey Miller and coworkers), and use of bacterial CRISPRs.

Shawn Burgess (National Institutes of Health, USA) described the use of high throughput sequencing to identify the sites of murine leukemia virus integration in the human genome. His lab constructed libraries using restriction enzyme digestion of gDNA in combination with ligation-mediated PCR to enrich and identify viral insertion sites. In particular, they studied insertion sites in two ENCODE cell lines, K562 and HepG2. They found an insertion site preference in promoter and enhancer regions with a particular preference for strong promoters/enhancers characterized by profiles of active chromatin marks.

Kevin Gunderson (Illumina, USA) described how transposase biochemistry is being used to streamline library preparation for high throughput sequencing and to create novel sequencing libraries supporting synthetic long read (SLR) technology. In particular, he presented recent results on an imputation-free SLR method that used Tn5 transposase to tagment (fragment and add adapters) the genome while maintaining contiguity information. Contiguity is preserved since transposases physically hold the fragments together until the protein is removed or denatured after compartmentalized dilution. These libraries were sequenced and haplotype phases reconstructed (imputation-free), with over 95% variants phased at a low switch error rate (1 in 10 Mb) and an average N50 contig length of over two Mb. This approach was also combined with 3 kb Mate Pair libraries to create assembly scaffolds of over 27 Mb. Such SLR technology approaches should greatly expand the utility of short-read sequencing by synthesis platforms for obtaining long-range information.

### Session 2: a transposon eye-view of primate evolution

Retrotransposon insertions generate both intra- and inter-individual genetic diversity in mammalian genomes. The talks in this session focused on exciting, unpublished discoveries in four major areas: 1) the identification of polymorphic retrotransposon insertions in primate genomes; 2) the discovery of somatic retrotransposon insertions in the human brain; 3) the identification of host mechanisms to restrict retrotransposition; and 4) the elucidation of mechanistic steps of L1 retrotransposition.

Mark Batzer (Louisiana State University, USA) identified polymorphic retrotransposons in primate genomes and demonstrated that their insertion rates among primates are relatively constant. However, he noted the striking exception of how Alu insertions have dramatically declined in the orangutan genome. Dr. Batzer then described how the polarity and homoplasy-free (the unlikely event where independent insertions occur at the same genomic site) characteristics of many polymorphic retrotransposon insertions allow their use as genetic markers to resolve phylogenetic relationships among human and non-human primates. Importantly, Dr. Batzer reported that approximately 4% of retrotransposon insertions among primates are not identical by descent, but instead represent closely spaced, parallel, independent insertions into a genomic locus. The identification of such events is of paramount importance to allow accurate use of polymorphic retrotransposons as genetic markers in phylogenetic studies.

Geoffrey Faulkner (Mater Research Institute, University of Queensland, Australia) described enhancements to a retrotransposon capture sequencing (RC-seq) protocol, and used them to uncover somatic L1 retrotransposition events in ‘bulk’ human brain samples and single hippocampal neurons. Dr. Faulkner uncovered greater than 300,000 somatic L1 insertions in hippocampal tissues, confirming earlier studies from his laboratory. He then used RC-seq to identify somatic L1 retrotransposition events in single hippocampal nuclei, and estimated that hippocampal neurons may contain approximately 15 somatic L1 retrotransposition events per cell. Dr. Faulkner also discussed preliminary RC-seq experiments conducted on genomic DNAs derived from frozen hippocampus and matched fibroblast samples from an Aicardi-Goutieres Syndrome (AGS) patient that harbor mutations in each allele of the *SAMHD1* gene. Once again, he identified somatic L1 insertions in the AGS brain tissue, though the relative numbers of insertions were not increased when compared to wild type controls. Together, these data provided compelling evidence that L1 retrotransposition continues to create somatic mosaicism in the human brain.

David Greenberg (Haussler laboratory, University of California, Santa Cruz, USA) described how the two human Krüppel-associated box zinc-finger proteins (KRAB-ZNF), ZNF91 and ZNF93, restrict the transcription of certain SVA and L1 retrotransposons and may protect primate genomes from unabated retrotransposition. The team found KRAB-associated protein 1 (KAP1/TRIM28) and human ZNF91 were enriched at SVA elements in mouse embryonic stem cell lines that contain a single human chromosome. Reporter assays demonstrated that ZNF91 inhibited SVA expression by binding to the SVA variable nucleotide tandem repeat sequence. Similar analyses suggested ZNF93 could bind within the 5′UTRs of evolutionarily older (PA3-PA6) L1 subfamilies. Notably, the ZNF93 binding sequence is absent from the evolutionarily younger (PA1 and PA2) L1 subfamilies, suggesting a mechanism for how younger L1s have evaded the repressive effects of ZNF93.

Pam Cook (Furano laboratory, National Institutes of Health, USA) showed that purified L1 ORF1p is phosphorylated on multiple residues. A subset of these residues was found to be critical for retrotransposition in HeLa cells. Remarkably, each residue in that subset lies within canonical target and docking motifs of proline-directed kinases (PDKs) and is highly conserved in both primates and mouse. Mutations of these residues inhibited L1 activity, but substitution with the phosphomimetic aspartic acid could restore L1 function. Thus, phosphorylation may represent an important mechanism to regulate L1 retrotransposition *in vivo*.

John Moran (HHMI/University of Michigan, USA) discussed preliminary data on the identification of sequences within L1 mRNA required for retrotransposition. The team replaced the 3′ poly (A) signal of an engineered human L1 with a sequence cassette derived from the 3′ end of a long non-coding RNA (MALAT1), which previously was shown by Wilusz and colleagues to form a stabilizing triple helical structure at the 3′ end of an mRNA. The resultant L1/MALAT RNAs accumulate in cells, serve as translation templates for the L1 encoded proteins, but lack a poly (A) tail and cannot undergo retrotransposition *in cis*. However, proteins translated from L1/MALAT mRNA could promote the retrotransposition of mutant L1 and Alu RNAs that contain poly (A) tails *in trans*. These data suggest that the presence of a poly (A) tail is required for efficient L1-mediated retrotransposition.

### Session 3: mechanisms of genome evolution I

This session focused on the different roles that mobile elements have played and will continue to play in genome evolution. The talks highlighted the diversity of contributions mobile elements make in genome evolution.

Marlene Belfort (University at Albany SUNY, USA) talked about group II introns that are the putative ancestors of both the spliceosomal introns and retrotransposons. She highlighted the absence of group II introns from eukaryotic nuclei and discussed the conundrum this implies with respect to their putative ancestral relationship to spliceosomal introns. She also talked about how retrotransposition is promoted by the synergistic relationship between the intron and the enzyme relaxase, whose gene is split by the intron.

Lynne Maquat (University of Rochester Medical Center, USA) spoke about mRNAs that are regulated post-transcriptionally via Staufen-mediated mRNA decay due to a STAU-binding site in their 3′UTR. Interestingly, she showed that in many cases these binding sites consisted of a 3′UTR Alu element or another type of SINE that base-paired with a partially complementary SINE within an lncRNA or another mRNA. She presented results that suggest a role for mobile elements in the intricate network of post-transcriptional interactions that regulate gene expression.

Scott Waddell, (University of Oxford, UK) reported on transposition events in the memory-relevant neurons in the *Drosophila* brain. He also showed that loss of piRNA proteins correlated with even higher levels of transposon expression in brain. He reported over 200 *de novo* transposon insertions in neurons through paired-end sequencing. His observations indicate that genomic heterogeneity is a conserved feature of the brain.

Kazufumi Mochizuki (Institute of Molecular Biotechnology, Austrian Academy of Sciences, Austria) presented results on the ciliated protozoan *Tetrahymena*, which is a model for programmed transposon-containing DNA elimination. The main analysis by high throughput sequencing involved measuring or analyzing the production and turnover of scan *RNAs (scnRNAs)* during the conjugation process. Notably, they found that there were two types of Internal Eliminated Sequences (IESs): those producing sncRNAs and those eliminated at a later stage potentially because of sequence similarity to the ones of the first type.

Guillaume Cornelis, (Institut Gustave, Roussy, France) talked about the exaptation during mammalian evolution of multiple retroviral envelope genes called *Syncytins* that function in placenta formation. He described three hitherto independent captures of endogenous retrovirus-derived envelope genes in Carnivora, Ruminantia, and Marsupial genomes. Together with previously reported cases, the data suggest that this exaptation process has been a major driving force in placenta evolution and diversity.

Finally, Guillaume Bourque (McGill University, Canada) discussed the impact that transposable elements have had on the evolution of human gene regulation. He showed that mobile elements have been a major contributor to host regulatory and transcript innovation, but also highlighted that these elements have been a significant source of ‘biochemical noise’ with limited impact on phenotype.

### Workshop 1: transposition mechanisms and regulation

Workshop 1 represented a mixture of TE structural studies, TE insertion targeting studies, and TE regulation by small RNAs. The first two talks shed light on key aspects of the protein dynamics involved in TE duplication.

Stuart Le Grice (National Institutes of Health, USA) presented the structure of the budding yeast Ty3 reverse transcriptase (RT). He showed that this protein is actually a dimer, but only in the presence of its polypurine tract DNA/RNA hybrid substrate. He demonstrated that the Ty3 RT structure is topologically highly similar to HIV-RT and XMRV-RT. However, a critical difference is that unlike other RT proteins, Ty3 RT adopts an asymmetric homodimeric structure when bound to its substrate and unlike dimeric retroviral enzymes, the DNA polymerase and RNase H catalytic centers of Ty3 RT reside in different subunits.

Orsolya Barabas (European Molecular Biology Laboratory, Germany) spoke about the critical need to understand bacterial conjugative transposons, as they are responsible for the transmission of antibiotic resistance. Dr. Barabas’s lab has determined the crystal structure of the conjugative transposon integrase enzyme from the vancomycin-resistance carrying *Tn1549* and demonstrated that this protein is active as a dimer and triggers formation of heteroduplex DNA intermediates during recombination.

The focus of Workshop 1 then changed to understanding the mechanism of the targeting of TE insertions. Jake Jacobs (Zaratiegui lab, Rutgers University, USA) described a model suggesting the integration of Tf1 retrotransposons in *Schizosaccharomyces pombe* is targeted away from genes and towards stalled replication forks. The Zaratiegui lab identified a strong correlation of Tf1 insertion at sites of Sap1 protein binding, which suggests Tf1 is directed to integration sites via the Sap1 protein. Jacobs showed that Sap1 binds DNA and creates a replication fork barrier. He used an elegant two-plasmid transposition assay to determine that Sap1 is important, but not necessary, for targeting Tf1 insertion, and that the fork barrier activity of Sap1 binding sites is an important secondary requirement for Tf1 transposition at stalled replication forks.

Next, Bao Ton-Hoang (Laboratoire de Microbiologie et Génétique Moléculaires, France) investigated the mechanism of targeting the replication fork by bacterial ‘HuH’ single-strand transposases encoded by the IS200/IS605 family. Ton-Hoang’s results suggested that the HuH transposase might be targeted to replication forks by direct interaction with structured DNA and that the interaction may be mediated by the single-strand DNA-binding proteins Ssb and RecA.

The final section of Workshop 1 focused on the regulation of TEs by small RNAs. Antoine Molaro (Fred Hutchinson Cancer Research Center, USA) showed that there are both *piwi*-interacting small interfering RNA (piRNA)-dependent and -independent mechanisms responsible for the TE epigenetic programming of mouse primordial germ cells. Dr. Molaro demonstrated that TE *de novo* methylation could be separated into two independent waves during germ cell development. The first wave appears unspecific and targets methylation genome-wide. However, some TE promoters escape this initial silencing and instead become the target of a piRNA-dependent secondary wave.

Sergey Shpiz (Institute of Molecular Genetics, Russia) discussed piRNA formation in the *Drosophila* germline. Dr. Shpiz demonstrated that new TE insertions into euchromatic regions of the genome become piRNA producing loci, and produce piRNAs from the surrounding genic sequences. Most of the insertions produced piRNAs from both sides of the insertion and from both strands. Importantly, Dr. Shpiz demonstrated these new genic piRNAs can regulate the mRNA levels of the genes from which they originated.

R. Keith Slotkin (The Ohio State University, USA) showed that genic mRNAs can be regulated by the small RNAs that TEs produce in Arabidopsis. Dr. Slotkin demonstrated that upon TE transcriptional activation the TE mRNAs are degraded into abundant small interfering RNAs. These siRNAs are incorporated into gene-regulating Argonaute proteins and function to regulate partially complementary genic mRNAs akin to a microRNA. In addition, Dr. Slotkin showed that TEs carry non-protein coding fragments that give rise to small RNAs that can target specific host genes and potentially favor TE propagation.

### Session 4: mechanisms of genome evolution II

David Kingsley (Stanford University, USA) summarized over a decade of work conducted in his laboratory to elucidate the genetic basis of ecological adaptation in stickleback fish. One of the lessons learned is that the molecular changes underlying the evolution of new morphological traits involve noncoding cis-regulatory sequences such as enhancers, rather than mutations in protein-coding sequences. In some recurrent instances, changes in regulatory elements include the deletion of a pre-existing enhancer or the co-option of transposable element sequences to form a novel enhancer. Kingsley argued that such cis-regulatory modifications also play a prominent role in the adaptation of other vertebrates, including humans, to rapid changes in their environment.

Daniel Barbash (Cornell University, USA) reported new results on the molecular mechanisms by which two ‘speciation’ genes normally function within *Drosophila* species. Genetic, genomic, and biochemical data suggest a model whereby the two encoded proteins from these speciation genes act together to suppress a broad range of repetitive elements in the *Drosophila* germline. Interestingly, these proteins also exhibit a gain-of-function phenotype in species hybrids that may contribute to transcriptional reactivation of previously silent transposons and chromosomal abnormalities.

Anne-Marie Dion-Côté (University Laval, Canada) presented data from another speciation model, the lake whitefish, supporting the notion that many transposable elements are massively activated transcriptionally in interspecific backcross hybrids of closely related species. There appears to be a positive correlation between the level of transposon transcript upregulation and the appearance of malformations or deformities in the backcross embryos, suggesting that hybrid breakdown in part may be caused by a failure to repress the transcriptional activity of transposable elements.

A presentation by David Kelley (Harvard University, USA) illustrated one of the potential mechanisms that could contribute to phenotypic consequences when the transcription of mobile genetic elements goes awry. He showed that transposable elements are frequently transcribed as part of long noncoding RNAs (lncRNAs) in various human cells. Transposon sequences embedded in lncRNAs are not biochemically inert but interact directly with a variety of RNA-binding proteins, as determined by cross-linking immunoprecipitation experiments followed by high-throughput RNA sequencing (CLIP-seq). Though it remains to be seen whether these interactions are relevant to cellular function, the data indicate that transposons are integral components of the intricate network of ribonucleoproteins found in human cells.

Cédric Feschotte (University of Utah, USA) explored the evolution and genomic impact of mobile DNA in yet another group of organisms, the bats. While bat genomes are relatively small and show little size variation among extant species, analyses of ten bat species representing five major chiropteran families suggest dramatic variation in transposable element activity among different bat lineages. Transposon expansions are counteracted by a high rate of DNA loss compared to other mammals and predominantly via large-scale deletion events, uncovering a previously underappreciated mechanism for genome size homeostasis.

### Session 5: the contribution of host factors to DNA mobility

Suzanne Sandmeyer (University of California, Irvine, USA) discussed research showing that the budding yeast, gypsy-like long terminal repeat retrotransposon Ty3 and its long terminal repeats act as pheromone-inducible promoters throughout the yeast genome. Work presented also showed that the RNP granules where Ty3 assembles have components in common with the animal germ cell granules that are known to be sites where RNA interference suppresses retrotransposons.

Jef Boeke (New York University Medical Center, USA) described studies of L1 host factors discovered through proteomic screens, including the UPF1 RNA decay protein and the PCNA sliding clamp. Studies suggest the PCNA is required after endonucleolytic cleavage of the target DNA. Preliminary studies suggesting cell cycle regulation of L1 through imaging and synchronization were also described.

Axel Horn (Tulane University, USA) presented data from yeast (Zorro3) and human (L1) non-LTR retrotransposons showing that an interaction with the ESCRT membrane budding complex plays an important role during the life cycle of LINE-like retrotransposons.

Alan Engelman (Dana-Farber Cancer Institute, USA) discussed allosteric inhibitors of the HIV-1 integrase that engage the catalytic core domain dimer interface at the binding site for the cellular LEDGF/p75 protein. The compounds induce integrase multimerization and compete for LEDGF/p75 binding. Interestingly, they were most potent during virus assembly, a point in the virus life cycle where LEDGF/p75 is apparently unable to shield the integrase from multimer induction.

Pascale Lesage (CNRS, Inserm, Institut Universitaire d’Hématologie, France) showed a functional interaction between Ty1 integrase and a subunit of Pol III, which is essential for Ty1 integration site determination, thus answering the long-standing question of why Ty1 integrates preferentially at genes transcribed by Pol III.

Mireille Bétermier (CNRS Centre de Génétique Moléculaire, France) studied the molecular mechanism involved in the precise excision of Internal Eliminated Sequences (IES) during assembly of the somatic genome of the ciliate *Paramecium*. She presented work showing that a prerequisite to IES end cleavage by *PiggyMac*, a domesticated *piggyBac* transposase, is the availability of a specific Ku heterodimer, which forms a complex with *PiggyMac* in cell extracts.

Nancy Craig (HHMI/Johns Hopkins School of Medicine, USA) presented work in yeast showing that the non-homologous end-joining (NHEJ) DNA repair pathway is required for rejoining of the donor site after excision of the *piggyBat* transposon but is not required for repair after transposon integration at the insertion site.

### Session 6: the biological impact of mobile DNA, friend or foe I

Shiv Grewal (National Institutes of Health, USA) presented his finding that in *S. pombe* heterochromatin formation can be induced at specific sites by environmental conditions. Regions of heterochromatin termed ‘Islands’ include meiotic genes and this heterochromatin forms independently of RNAi factors. Other clusters of heterochromatin termed ‘HOODS’ include meiotic genes and retrotransposons but formation of this heterochromatin relies on RNAi factors. Grewal reported that the heterochromatin in Islands and HOODS both rely on an RNA processing network that includes a core module (MTREC) composed of Mtl1 and Red1. MTREC degrades meiotic and retrotransposon mRNA, and through interactions with Nrl1 and splicing factors, recognizes cryptic introns and creates heterochromatin, whereas Mtl1 and Ctr1 promote proper processing of intron-containing telomerase RNA to maintain telomeres.

Michelle Longworth (Cleveland Clinic, USA) presented a new mechanism of silencing retrotransposable elements in *Drosophila* that requires the condensin II subunit dCAP-D3. This protein plays a key role in genome integrity by inhibiting double strand breaks in transposable elements. 3D chromatin data indicated that dCAP-D3 causes the formation of rigid chromatin loops that exclude transposable elements. This organization of chromatin inhibits the expression of retrotransposons and reduces homolog pairing of chromosomes that leads to double strand breaks.

Damon Lisch (University of California, Berkeley, USA) described his studies of the diverse family of Mutator elements in maize that rely on the autonomous element for transposition activity. He reported the discovery of Muk, a Mutator element in maize that, as the result of a rearrangement, produces a hairpin transcript that silences *MuDr*. This mechanism involves the DNA methylation and histone modification of promoter sequences. The silencing of *MuDr* is relieved in young leaves but returns during specific stages of development. Lisch proposed an intriguing model arguing that maize turns off its defense against transposable elements temporarily in leaves to allow the transmission of newly produced *MuDr* small RNAs into the germline where they can repress transposition.

Axel Imhof (Ludwig Maximilians University of Munich, Germany) presented evidence for a unique system of reproductive isolation in *Drosophila* that is proposed to cause speciation. Lhr and Hmr form a centromeric complex that mediates chromosome segregation and inhibits expression of transposable elements. *D. melanogaster* and *D. simulans* express different levels of Lhr and Hmr. In species hybrids, changes in the Lhr to Hmr ratio cause extensive mis-localization of the complex, increased expression of transposable elements, and impaired cell proliferation. These results argue that centromere binding proteins can play a surprising role in generating biodiversity.

Henry Levin (National Institutes of Health, USA) presented results that test Barbara McClintock’s hypothesis proposing that transposable elements benefit the host by rearranging the genome in response to stress. The LTR retrotransposon Tf1 of *S. pombe* integrates into the promoters of stress related genes. Importantly, Tf1 increases the expression of adjacent genes in 40% of the insertions tested. The impact of Tf1 integration on growth in conditions of stress was examined with cobalt chloride. Large cultures of cells with diverse profiles of Tf1 insertions show that integration in specific intergenic sequences allows these cells to out compete others when cobalt chloride is present. These results argue that Tf1 integration possesses specific features that result in resistance to cobalt chloride and provide evidence supporting McClintock’s hypothesis.

### Session 7: the biological impact of mobile DNA, friend or foe II

William Theurkauf (UMass Medical School, USA) discussed the adaptive genome defense mounted in *Drosophila* by piRNAs that silences transposons during germline development. He showed evidence that the HP1 homolog Rhino directly binds to the heterochromatic piRNA clusters and triggers piRNA production, leading to silencing of other sense and antisense transcripts. He also presented data indicating Rhino might anchor a nuclear complex that suppresses piRNA cluster transcript splicing, which may be required to differentiate piRNA precursors from mRNAs. The *Drosophila* findings were quite reminiscent of a theme introduced earlier in the meeting by Shiv Grewal (NCI, NIH) from his work in *S. pombe*, in which he also concluded that stalling splicing might be sufficient to induce the production of siRNAs.

Continuing the theme of adaptive RNA-guided defenses, Luciano Marraffini (Rockefeller University, USA) discussed how prokaryotic CRISPR-Cas immune systems might distinguish pathogenic from self and commensal elements. He discussed temperate phages that can integrate into the bacterial chromosome and could be considered ‘commensals’ of bacteria because they can carry genes that provide a fitness advantage to their host. Luciano elucidated an elegant means by which CRISPR-Cas might tolerate beneficial temperate phages through transcription-dependent DNA targeting, leading to no action against lysogenic prophages but targeting during the prophage lytic cycle. His results highlighted the myriad of means used by defense systems not just to distinguish ‘self from non-self’ but also to encode ‘tolerance to non-self’ in this exciting prokaryotic branch of adaptive immunity.

Grace Wyngaard (James Madison University, USA) reported on genome elimination in the somatic cells of copepods. Germline genomes in copepods can be 5-75X larger than somatic cells, due to a massive elimination of mobile elements from the presomatic cell lineage. She raised the speculation that the billions of basepairs of excised DNA might serve an adaptive role that provides the embryo with much of the DNA material needed to replicate the genome during subsequent cleavage divisions until the embryo hatches into a feeding larvae. Both the phenomenal genome diminution and the speculation that this could be adaptive resulted in much discussion among the meeting participants.

Todd MacFarlan (National Institutes of Health, USA) discussed his work on the intriguing hypothesis that some members of the large and rapidly evolving KRAB-ZFP transcription factor family might function to silence endogenous retroviruses (ERVs). He showed that the mouse KRAB-ZFP protein Zfp809 binds and silences a sub-group of class I ERVs that utilize a proline primer binding site (Pro-PBS) via recruitment of heterochromatin establishment machinery. This work extends previous findings from Steve Goff’s lab, which originally identified Zfp809 in a biochemical screen for repressors of murine leukemia virus.

Vasavi Sundaram (Washington University, St. Louis, USA) presented data on comprehensive mapping of binding sites for 26 pairs of orthologous transcription factors (TFs) in human and mouse cell lines. She presented data that on average 20% of TF binding sites were within TEs. TF binding peaks derived from TEs had epigenetic signatures consistent with regulatory activity. A substantial fraction of TE-derived TF binding events showed cell type-specificity. The results significantly extend previous findings that TEs have continuously shaped gene regulatory networks during mammalian evolution by their widespread contribution of TF binding sites.

Harmit Malik (HHMI/Fred Hutchinson Cancer Center, USA) presented work from his lab on using evolutionary signatures of genetic conflicts in host-virus ‘arms-races’ to dissect the basis of differential viral susceptibility between different closely related host species. He presented findings about the antiviral protein MxA, which is remarkably broad in its antiviral range despite having to specifically recognize diverse, unrelated viral proteins in order to mount its antiviral action. In an analysis of primate MxA proteins, he showed that there were significant clusters of positive selection, which may each have specialized to be recognition interfaces for proteins from unrelated viruses. He concluded by suggesting that signatures of genetic conflicts may also allow the specific dissection of host defenses against their resident mobile elements.

### Workshop 2: genetic variation and evolution

Peter Atkinson (University of California, Riverside, USA) reported the identification, of a new active Mutator superfamily transposon from the mosquito *Aedes aegypti* called *Muta1*. The authors cloned this element from the genome and showed it is transpositionally active in a range of insect species, including in its own host, in which it can be used to achieve genetic transformation.

Csaba Miskey (Paul Ehrlich Institut, Germany) described a primate gene called SETMAR that emerged from the fusion of a histone methyltransferase SET-gene and the transposase of a *mariner* transposon. Miskey and colleagues used chromatin immunoprecipitation experiments to map more than two hundred SETMAR-bound loci in the genome of human cells. The presence of SETMAR at its binding sites was associated with increased levels of local H3K36 di-methylation due to the methyltransferase activity of the SET-domain. These results support the hypothesis that the evolutionary recruitment of SETMAR’s transposase domain resulted in the emergence of an anthropoid-specific gene-regulatory network.

Keizo Tomonaga (Kyoto University, Japan) focused on endogenous Bornaviruses, which are non-segmented negative strand RNA viruses that have left their own endogenous elements in the genomes of many animal species. Tomanaga demonstrated that mouse endogenous bornavirus-like elements (EBL) produce the pachytene piRNAs in the testis, and also demonstrated that an EBL element in ground squirrel could efficiently inhibit the replication of exogenous bornavirus in cultured cells. This talk provided insights into the interaction between RNA viruses and hosts but also the biological significance of endogenization of non-retroviral viruses.

Josefa Gonzalez (Institut de Biologia Evolutiva, CSIC-Universitat Pompeu Fabra, Spain) reported the molecular mechanism underling the phenotypic effects of two putatively adaptive transposable elements discovered in *Drosophila melanogaster*. A *Bari1* insertion was reported to mediate resistance to oxidative stress by adding *CncC* transcription factor binding. And a pogo insertion was found to increase expression of *CG11699*, which leads to increased *ALDH-III* enzymatic activity and resistance to xenobiotic stress. These results contribute to our understanding of eukaryotic stress response and highlight the role of TEs in environmental adaptation.

Inigo Narvaiza (Salk Institute for Biological Studies, USA) and colleagues investigated the function of L1s in induced pluripotent stem cells (iPSCs) from human and non-human primates (NHPs). They used iPSCs from humans, chimpanzees and bonobos to explore factors that could have contributed to primate evolution and genomic diversity. Comparative gene expression analysis of human and non-human primates (NHP) iPSCs revealed differences in the levels of APOBEC3B (A3B) and PIWIL2, two regulators of L1 transposition. Higher levels of A3B and PIWIL2 in human iPSCs correlated with decreased L1 mobility compared to NHP iPSCs. These results were consistent with an analysis finding increased copy number of species-specific L1 elements in the genome of chimpanzees compared to humans.

Liana Fasching (Jakobsson lab, Lund University, Sweden) presented data demonstrating that transcription of ERVs in neural progenitor cells is dynamically regulated and activation of ERVs resulted in increased expression of nearby genes as well as the production of novel long non-coding RNAs. These data suggest that ERVs play a role in controlling genetic networks in neural progenitor cells. Since elevated levels of ERVs have been found in several neurological disorders, further studies on the role of ERVs in the control of gene expression are warranted in both the healthy and diseased brain.

Finally, Josh Dubnau (Cold Spring Harbor Laboratory, USA) discussed the potential role of retrotransposons in neurodegeneration and age-related cognitive decline. He showed evidence that a diverse set of retrotransposons is transpositionally active in specific sets of post-mitotic neurons and glia in the fly brain. Some of these retrotransposons become aggressively active with age, leading to accumulation of *de novo* insertions in neurons. Dubnau and colleagues also demonstrated that genetically activating LINE-like and gypsy retrotransposons by disruption of the *Drosophila argonaute-2* gene, leads to rapid age-dependent memory impairment, defects in locomotion, and ultimately to shortened lifespan. Finally, Dubnau showed evidence that links this transposon activity to the neurodegenerative effects seen with TDP-43 pathology both in flies and in mammals, including humans. These findings have implications for the mechanisms of neurodegeneration seen in amyotrophic lateral sclerosis and frontotemporal dementia, where the TDP-43 protein’s pathology is central.

### Session 8: the interface between mobile DNA, DNA replication, and DNA repair/the molecular face of transposition

In the final session of the meeting, three talks showcased the versatile nature of the transposase family used by DNA transposons. A fourth talk focused on an interesting enzymatic collaboration between serine recombinases in site-specific recombination.

Rasika Harshey (University of Texas, Austin, USA) presented work on an under studied area of transposition involving the repair of DNA following transposition with bacteriophage Mu. Mu uses transposition as a mechanism for replication. During the lytic cycle, integration is via a ‘cointegrate’ mechanism where replication of the element is primed from the target DNA. However, when forming a lysogen in a new host, repair at the ends of the element and removal of the ‘flaps’ of DNA from the previous host occurs by an unresolved mechanism. Previous work indicated that, unexpectedly, host double-strand break repair was involved in flap removal. In surprising new work, evidence was presented indicating that the replisome involved in normal chromosomal DNA replication is actually responsible for repair. A model was presented attempting to reconcile this finding with what we know about bacteriophage Mu and other transposable elements.

Fred Dyda (National Institutes of Health, USA) presented work on the architecture of the Hermes transposase, a member of the *hAT* superfamily of DNA transposases. A crystal structure of the Hermes transposase complex with transposon end DNA sequences was presented. The transposase in the crystal structure is an octamer, a tetramer of four dimers, an oligomerization state that was confirmed both by electron microscopy and small-angle X-ray scattering technique. Biochemical data suggested that the octamer is needed to provide an array of DNA binding domains to find Hermes’ ends and to give sufficient binding affinity. Analysis indicated that the transposon ends are situated in a configuration such that the 3′-OH ends of the transferred strands orient to the suitably charged cavity in the complex where the target DNA likely resides. Further DNA-transposase crystal structures indicated that the configuration is consistent with double-strand break formation during excision by flanking-hairpin formation via a strand-switching mechanism. It is also consistent with the 8-bp duplication found *in vivo* at the sites of integration.

Phoebe Rice (University of Chicago, USA) presented work on a pair of large serine recombinases that are responsible for the integration of the SCC*mec* element encoding methicillin resistance in *Staphylococcus aureus*. Unlike more typical site-specific recombinases where a single recombinase recognizes a symmetric target site, SCC*mec* uses two serine recombinases that collaborate to recognize an asymmetric site. This asymmetry may have broadened the options for evolving an integration site given that site-specific recombinases that use a single enzyme are constrained to inverted repeats found naturally in the host. Preliminary work also gave provocative possibilities for other proteins encoded on the element.

Joseph Peters (Cornell University, USA) presented work on Tn7, an element that is notable because of the level of control it has in target site selection and the broad diversity of bacteria that it uses as a host. The element uses five transposon-encoded proteins, including a heteromeric transposase (TnsA and TnsB), a regulator protein (TnsC), and target site selecting proteins that can choose a single site found in bacterial chromosomes (via TnsD) or target a complex found during lagging-strand DNA replication (via TnsE). TnsE was previously shown to recognize DNA replication by associating with 3′ recessed ends and the sliding clamp processivity factor. Current work suggests that TnsE adapts to the host sliding clamp protein and that this adaptation provides species specificity to Tn7-like elements. Bioinformatics analysis was presented indicating that elements with heteromeric transposases are more widespread than previously appreciated and have adapted new target site selection programs distinct from Tn7-like elements.

Understanding transposition and its interaction with host genomes will not only explain the host response to the environment but also help to reveal its concealed role in evolution. The results presented at this conference show that much progress has been made in achieving these goals.

### St Malo 2016

The tradition of a large international meeting on ‘mobile DNA’ will continue. In the spring of 2016 (16 April to 19 April), the International Congress on Transposable Elements will be held in St Malo, France. Stay tuned for more details!

## Abbreviations

siRNAs: small interfering RNAs
TEs: Transposable elements
L1: LINE-1
hiPSCs: human induced pluripotent stem cells
RC-Seq: Retrotransposon capture-sequencing
DGRs: Diversity Generating Retroelements
SLR: synthetic long read
AGS: Aicardi-Goutieres Syndrome
KRAB-ZNF: Krüppel-associated box zinc-finger proteins
KAP1/TRIM28: KRAB-associated protein 1
PDKs: proline-directed kinases
IESs: Internal Eliminated Sequences
RT: reverse transcriptase
piRNA: *piwi*-interacting small interfering RNA
lncRNAs: long noncoding RNAs
NHEJ: non-homologous end-joining
*scnRNAs*: scan *RNAs*
CLIP-seq: cross-linking immunoprecipitation experiments followed by high-throughput RNA sequencing
ERVs: endogenous retroviruses
TFs: transcription factors
EBL: endogenous bornavirus-like elements
iPSCs: induced pluripotent stem cells
NHP: human and non-human primates.

